# Physician View and Experience of the Diagnosis of Autism Spectrum Disorder in Young Children

**DOI:** 10.3389/fpsyt.2019.00372

**Published:** 2019-05-28

**Authors:** Delphine Jacobs, Jean Steyaert, Kris Dierickx, Kristien Hens

**Affiliations:** ^1^Center for Biomedical Ethics and Law, Department of Medicine, KU Leuven, Leuven, Belgium; ^2^Center for Autism Expertise, Child and Adolescent Psychiatry, University Hospitals Leuven, Leuven, Belgium; ^3^Department of Neurosciences, KU Leuven, Leuven, Belgium; ^4^Department of Philosophy, University of Antwerp, Antwerp, Belgium

**Keywords:** autism spectrum disorder, diagnosis, physicians, interviews, experiences, views, clinic, integration

## Abstract

**Introduction:** Clinicians working with children with autism spectrum disorder (ASD) occupy an important position between parents and the wide-ranging research findings. However, it is not widely known how clinicians view and experience ASD in children, even though their perspective has been shown to significantly influence their work.

**Material and methods:** Sixteen physicians working with preschool children without a diagnosis of (intellectual or other) disability with a (presumed) diagnosis of ASD participated in a semistructured interview. They described their professional views on ASD, and how they experienced its use in their clinical practice. The data were analyzed by applying the qualitative research method of interpretative phenomenological analysis of the data through Nvivo 11.

**Results:** The main topic of the interviewed physicians’ views and experiences of ASD in a young child comprised three inductively established themes: 1) physicians’ views on ASD are multifaceted but fit within their personal clinical styles; 2) the ASD diagnosis is a “descriptive” part of a clinical trajectory; and 3) ASD treatment is a mix of “standard” approaches and a personalized search. These physicians’ perspectives on ASD are composed of multiple and sometimes ambiguous facets. However, their views are embedded in their personal clinical styles in general (i.e., beyond ASD) and are experienced as clinically “workable.” With the aim of finding an adequate approach to the problems parents bring to their office, many interviewed physicians say that—complementary to or rather than a classificatory diagnosis—they prefer using a personalized “profile” of a child in a therapeutic “process.”

**Conclusions:** The interviewed physicians consider doubts and concerns to be an inherent part of their clinical work with ASD in young children, but do not experience this ambiguity as an obstacle to clinical care. These physicians deal with the multiplicity of their views on ASD by basing their eclectic views on their generally adopted clinical styles, and by selecting what works for them, and for the parents and child, from what they regard as the ‘textbook knowledge’ on ASD. We discuss the implications of these findings for translating research results to the clinic.

## Introduction

Autism spectrum disorder (ASD) is a heterogeneous disorder, in its phenotypical presentation and when it comes to findings concerning cause, treatment, and prognosis (1–3). In the last five decades, there has been a significant rise in the estimated diagnostic prevalence of ASD, from 5 children in 10,000 in the 1960s (4), to 1 in 59 8-year-old children today in the U.S. (5). ASD is a neurodevelopmental condition, characterized by early-onset difficulties in social communication, and restricted, repetitive behaviors, and narrow interests (6). It is diagnosed clinically, preferably in a multidisciplinary assessment assisted by specific diagnostic tests (7, 8). Kanner’s nosological entity of “autistic disturbance of affective contact” evolved together with Asperger’s “autistic psychopathy” to the contemporary diagnosis of ASD (9, 10). Philosophical and historical research shows that autism is not unequivocally defined and is understood in different ways throughout times and cultures (11, 12).

It is unclear in what way an—sometimes contradictory—array of research findings can be and is being translated into information that is useful and meaningful to clinicians. Indeed, after three quarters of a century of research (13) and clinical experience, there is a consensus that ASD is heterogeneous in all its aspects (14–16), and that relatively little is known about ASD—its causes, its correlates, and its consequences (13). For example, the investigated and reported heritability of ASD is highly variable, ranging from 50% to more than 90% (17–19). Concerning ASD intervention, progress has undoubtedly been noted, but a long journey remains ahead (17, 20).

A growing gap is argued to exist between “basic” ASD science on the one hand and clinical practice and the community on the other (13, 15). Psychiatry’s quest for greater scientific recognition might be more supportive of psychiatric research than of clinical practice (21). An overview of recent findings on early ASD diagnosis and intervention concludes that work remains to be done to ensure that research findings are translated into clinical practice (22). Indeed, the movement from science to practice is a challenge and an important next step for the field (3). In this translation, a clinical approach inspired by findings from “basic research” on ASD (13) may be only partially helpful in responding to parents’ worries about and problems with a child mildly impaired by ASD-like behavioral problems (23–26). This demand for a transfer of research findings to clinical practice is replicated by the autism community’s need for the autism researcher (and also the clinician, undoubtedly) to make every possible effort to translate knowledge about ASD to a lay audience (27).

Besides this translation issue, although the diagnostic criteria in the *Diagnostic and Statistical Manual of Mental Disorders* (*DSM-5*) and the *International Classification of Diseases* (*ICD-11*) are articulate, it is still unclear how physicians professionally define and understand ASD (11, 12). At the same time, physicians’ views have been shown to influence their work significantly (28–30). Consequently, how clinicians actually view and experience ASD, its use, and usefulness in their practice is important to know, yet has rarely been studied (31). In a literature review of the research on parents’ and clinicians’ conceptualizations of ASD^1^, we identified nine studies on parents, five on clinicians and four on both parents and clinicians, published between 1993 and 2016. The papers’ disciplinary perspectives and research questions were extremely diverse. Most studies on clinicians actually did not focus on clinicians’ conceptualizations [for the two exceptions, see Refs. (32, 33)]. They mainly queried clinicians on a related topic like on how physicians viewed the barriers to screening, and decided on an ASD diagnosis (34) ^1^. Our review revealed that many clinicians had biopsychosocial understandings of ASD but were expressing biomedical views to patients. The included papers often reported on parents’ experiences of the psycho-relational impact of a diagnosis on child and parents, but did not report on clinicians’ experiences of this impact.

We aim to take a step toward filling this gap. Thus, the goal of this qualitative study is to explore and gain an insight into the conceptualizations of ASD of physicians working with preschool children without a diagnosis of disability. This aim was broken down into two main objectives: 1) physicians’ experiences of the psycho-relational impact of an ASD diagnosis; and 2) physicians’ views of ASD and an ASD diagnosis^2^. The results belonging to the former research question showed that physicians experience several important psycho-relational implications of an ASD diagnosis besides clear treatment-related consequences (35). Here we present the results answering the latter research question, exploring physicians’ views of ASD and an ASD diagnosis.

## Material and Methods

The material and methods used in this study have already been described in our article on the first half of the study results, answering the first research question (35).

### Sample

Although many kinds of healthcare professionals work with children with a (presumed) diagnosis of ASD, we chose to include as participants a select group of physicians in Flanders, Belgium, who are experienced in working with particular children. This interview study only included physicians who were experienced^3^ in working with children younger than 6 years of age, without an intellectual disability (ID) and other disability, and with a diagnosis of ASD or who displayed ASD-like behaviors that led parents to ask for an ASD diagnostic assessment. The group of physicians working with these children is of particular interest, because the increase in prevalence of the diagnosis in the last decade is partly attributed to children without comorbid ID being more frequently diagnosed (6), and because recently there has been a growing emphasis on diagnosing children with suspected ASD early on (36). The physicians we included are involved at an early stage of the assessment and treatment of young children with a (presumed) diagnosis. There are specific ethical questions that arise concerning the use of an ASD diagnosis in young children. For example, there is debate about whether research should focus on identifying cures for early-diagnosed ASD (13), and questions can be asked about the ethical implications of the only moderate stability of an early ASD diagnosis in preschool children (37). Since these children were not intellectually or otherwise disabled, the diagnosis of another disability would arguably not “overshadow” a physician’s experiences of the diagnosis of ASD in these children. We confined our sample to the profession of physicians in order to obtain a homogeneous group (38), and because in Flanders, a physician is necessarily a part of the multidisciplinary team performing ASD assessments. Such a team performs a clinical assessment in different developmental domains, most often assisted by diagnostic tests—most of which are evidence based, like ADOS (Autism Diagnostic Observation Schedule) and WPPSI-R (Wechsler Preschool and Primary Scale of Intelligence–Revised) (39, 40). Concerning postdiagnostic support, Flemish physicians may provide coordination of the professional help (especially at school), parent guidance, developmental follow-up of the child, and psychotropic drug prescription and follow-up; often they delegate (parts of) this support to other clinicians. Physicians were purposively sampled as potential participants (41). They were contacted by e-mail through two professional physicians’ organizations: the Flemish Association of Child and Youth Psychiatrists (VVK) and the Flemish Association of Disability Physicians (VVAG). We chose these two organizations, because for children in Flanders younger than 12 years, ASD is most often diagnosed in those who visit specialized outpatient clinics (58.2%), or rehabilitation centers (21.4%), or who are seen by child psychiatrists (13.3%) and pediatricians (2.0%)^4^ (42). Fourteen physicians spontaneously responded to our e-mail (sent to several hundreds of members of the two organizations), agreeing to be interviewed. After these 14 interviews were completed, we supplemented the sample of 14 volunteering physicians by explicitly inviting two more child neurologists to participate. This was done in order to broaden the data set to include experiences of the different types of physicians who regularly work with these children in Flanders ^5^, and to reach a consensus between the views interviewees expressed. The latter goal of saturation (43, 44) was checked collaboratively by the first and the last authors (DJ and KH). Thus, the final purposive sample comprised 16 physicians (45), of whom 9 were child psychiatrists, 4 were child neurologists, 2 were disability physicians, and 1 was a pediatrician. They were working in centers for developmental disorders, hospitals, private practices, and other work settings. Twenty-five percent of the participants was male, and their median age was 45. Information about the study was given orally and in written form, and all persons gave their written informed consent prior to their inclusion in the study, and to the publication of the study results. The Ethics Committee of the University Hospitals Leuven approved the protocol of this study on 3 February 2017 (Belgian Registration Number B322201731147). The study was performed in accordance with the ethical standards laid down in the 1964 Declaration of Helsinki and its later amendments.

### Data

Qualitative data were gathered using semistructured one-to-one interviews. We based the topic guide (**Table 1**) for the in-depth interview on our findings from a literature review on how clinicians conceptualize ASD (46) ^1^.

**Table 1:** Interview guide.

Research question: How do physicians view and experience ASD and an ASD diagnosis and its impact on children and parents?
1. Can you describe what ASD means to you? How do you *understand* ASD? What are the *consequences* of talking/thinking about ASD in the society in general? Both “positive” and “negative”?
2. What do you think and feel before, during, and after a diagnostic ASD assessment? Can you describe what you think and feel *before* a diagnostic ASD assessment? Can you describe what you think and feel *after* a diagnostic ASD assessment? Can you describe what you think and feel *during* a diagnostic ASD assessment?
3. Can you describe what “receiving an ASD diagnosis” means to you? How do you *understand* receiving an ASD diagnosis for child/parents? What, in your experience, are the *consequences* of an ASD diagnosis? What, in your experience, is the *impact* of an ASD diagnosis on the life of child/parents? Does the life of child/parents change? What exactly changes? What, in your experience, is the *impact* of an ASD diagnosis on how you/child/parents look at the child? Your look at the child? The look of the child at him/herself? The look of the parents at the child? Does something change, what exactly?
Are there things that are important to you that have not yet been addressed or things that I have forgotten to ask? Would you like to add something or change an answer?

ASD, autism spectrum disorder.

Since we used the qualitative method of *interpretative phenomenological analysis* (IPA) (47), the interviews focused on gathering information about how physicians view and experience ASD and an ASD diagnosis professionally. In expressing their views and experiences, physicians were invited to describe both emotions and thoughts. Indeed, the content of one’s thoughts contributes to the phenomenological experience of emotion, and accounts for differences in the experience of cognitively complex emotions (48). The interview guide consisted of open-ended questions. It was used openly and flexibly, in order to encourage the participants to elaborate freely and reflectively on a theme they wanted to discuss, even if that theme was to be covered later in the interview. Regardless of this, the same content was covered with each participant. At the end of the interview, the participants were asked if they wished to express anything else about the topic. All interviews were conducted in Dutch by the first author (DJ) and audio-recorded. Data were collected between July 2017 and February 2018. The interviews mostly took place at the participant’s office, a few times at the researcher’s office, or in the participant’s home. Interviews lasted for 40 to 100 min. In only two cases, the interviewer noticed time pressure affecting the interviewee; participants were free to say more on an interview topic if they chose to, and some physicians used more time than others to elaborate and clarify their experiences.

### Analysis

The data were analyzed using the procedures outlined for IPA (47). Briefly, IPA employs inductive analysis, grounded in the material, and in the second place a dialogue of the resulting findings with already existing theories. Smith et al. talked of a “double hermeneutic” in which the interviewees give interpretative narratives of their experiences, and the researcher analyzing these narratives interprets them when identifying themes that belong to the domain of the research question (38, 47).

DJ and the last author (KH) listened to the recordings independently. DJ transcribed each recorded interview verbatim, and then systematically and inductively coded the transcripts line by line in NVivo 11 (QSR International, 2017). The goal was first to identify thematic passages of text across the data of one interview. Consequently, a comparison was made across interviews. The analysis was collaboratively conducted by DJ and KH. In addition, all authors met regularly to discuss the interviews and the codes. The primary codes were clustered into recurrent subthemes and synthesized into themes (**Table 2**). An inductive analysis of the material entails that topics only tangentially related to the research question may emerge, like in this study many physicians talked about the connection between their views on an ASD diagnosis and on ASD treatment (43). Finally, an overall narrative account was created through a group analysis of the different interviews. Representative quotations were translated verbatim from Dutch to English, in order to illustrate the original expressions. Square brackets indicate information added from the interview material before or after the quote in order to give context for the quote.

**Table 2:** Physicians’ view on and experiences of ASD and an ASD diagnosis.

Themes	Subthemes
Physicians’ views on ASD are multifaceted but fits in their personal styles	View is implicit and multiple For one physician Between physicians No systematic difference between different specialisms
	View fits within personal clinical style
	View is “workable” View does not disturb clinical work ASD is a neurological reality ASD is heterogeneous, but characteristic (IQ + “real/nuclear” autism)
	Difficult integrations Clinic vs. training and research literature Medical training vs. psychotherapy training External demands vs. quality of care
The ASD diagnosis is a descriptive part of a clinical trajectory	“Process-diagnostics,” descriptive “profile” ASD difficult to define/diagnose, facts unclear Diagnosis contextual and multidisciplinary
	Parents important to diagnosis/phenotype/prognosis, potential communication through child’s behaviors
ASD treatment is a mix of “standard” approaches and a personalized search	Adherence to perceived “standard” approach
	“Standard” approach also useful for other children
	Supplemented with personalized search
	Parents’ experience and child’s communication

Several strategies were employed to strengthen the validity of the findings. First, the authors frequently met and discussed the data and their analysis. Second, the research project was subjected to peer scrutiny by researchers from the local psychology faculty and the pedagogy faculty during several meetings and seminars in which the anonymized interviews and the analysis were discussed. Finally, the researchers continually maintained a reflexive stance (49). These deliberative procedures enhanced the trustworthiness of the findings.

## Results

We will define view and experience as every perspective a physician expresses on ASD: its ontological understanding, phenotype, etiology, diagnosis, treatment, and prognosis. The inductively coded material on these different topics was interpretatively synthesized into three themes: 1) physicians’ views on ASD are multifaceted but fit within their personal clinical styles; 2) the ASD diagnosis is a “descriptive” part of a clinical trajectory; and 3) ASD treatment is a mix of “standard” approaches and a personalized search. We will discuss these themes and their subthemes consecutively.

### Physicians’ Views on Autism Spectrum Disorder Are Multifaceted But Fit Within Their Personal Clinical Styles

Within this first theme on physicians’ views on ASD, we will present four subthemes: 1) view is implicit and multiple; 2) view fits within a personal clinical style; 3) view is “workable”; and 4) difficult integrations.

#### View Is Implicit and Multiple

A physician’s expressions of his/her view on ASD are dispersed throughout the interview. What we extracted as components of a physician’s view on ASD is never explicitly expressed: it is always implicit and difficult to grasp. Moreover, this view is often not coherent and consistent. It is usually multifaceted: it consists of multiple, diverse, and ambiguous perspectives. For example, concerning the phenotype, the following physician says that every child with a diagnosis of ASD is different:

For me, these are all different children, I believe they all are different (physician 2).

However, later she enumerates several things she experiences as characteristic in children with ASD:

…if they leave, they always put their chair very neatly under the table … they have some ‘norm awareness,’ their conscience is usually quite okay. (physician 2).

Also, the multiplicity *between* different physicians’ views on ASD shows. For example, when it comes to prognosis, one physician views ASD as a dynamic (i.e., changing) condition, leading to easier psychotherapeutic work. Another physician views ASD in a more static way, leading to a treatment approach that can include medication.

Since I view autism in a more dynamic way, I feel that psychotherapeutically I can work more easily with these parents” (physician 4)

“It is good to know a child has autism, so I can make things more predictable for her and sometimes you also have to give medication.” (physician 5).

Moreover, physicians with a different medical training (child neurology, child psychiatry, disability medicine, pediatrics) cannot be distinguished by their—often multifaceted—view. The following child neurologist says she shares with parents her view on ASD as a dynamic condition:

I give them this message: it is possible we don’t give the diagnosis at 4 or 5 years, but the ‘auti-approach’ is very helpful now, even if he doesn’t have autism later on. I tell them it is a framework within which we help the child, but the diagnosis, we’ll see … (physician 15).

In the following quote, a child psychiatrist also favors a dynamic view, both for herself and for sharing with parents:

Since I handle it in a more dynamic way, I notice the therapy runs more smoothly, everything is easier, it is easier to remain “in process” with these parents if I explain it in a dynamic way. (physician 4).

This example illustrates how a different medical training does not necessarily entail a different view on ASD.

#### View Fits Within a Personal Clinical Style

The physicians’ multiple views on ASD are in line with their personal clinical styles or attitudes *in general*, i.e., their approach toward and perspectives on the children and parents they meet in their office *in general*. The following physician explains her view on treatment for children with problems in general, and later in the interview, she sets out a similar view on children with an ASD diagnosis:

In fact, doing what a child needs and attuning to that, I think, which will be different in each situation.

And later on in the interview, concerning ASD:

Of course, you can also—separate from the parent training [on ASD] that is fairly general, although there is a lot of possibility for questions—you can again look for each child: does she need a behavioral program or a signaling plan or a ‘traffic light,’ you name it, you can adapt these measures to each child’s needs. (physician 5).

As such, ASD is not approached all that differently from other behavioral diagnoses: ASD fits within the broader personal clinical style of a physician. For example, a physician says:

The Dialogue Model is such a nice way of conducting feedback sessions. But not only for autism! It is mapping the child, separately from a diagnosis, from the pure label. (physician 10).

So, she says that she has a general way of conducting feedback sessions in which the diagnosis is not central.

#### View Is “Workable”

A physician’s view on ASD is often multifaceted and ambiguous and never explicit. Nevertheless, their resulting fragmented view on ASD is always experienced to be *workable*: a physician appears to be able to use his/her tension-prone understanding of ASD when performing his/her clinical work. The fact that ASD is understood by no one as a clear and uniform notion, does not interfere with his/her clinical work. The following physician is asked if she feels the parents’ view on their child changes once the child receives a diagnosis: she feels she can transmit her view on ASD to parents and acquire a workable shared understanding with them:

I try not to change the parental image of the child too much. I ask the parents: “Look at your child, as he is now.” And if things change, I hope this is because parents understand a number of things, not because they feel like: “Our child is a real ‘autist’ now.” That’s what I try. (physician 3).

Besides not disturbing physicians, this tension-prone view on ASD does not prevent the interviewed physicians from experiencing ASD as self-evident. No one denies the neurological reality of a certain ‘autistic condition’ and the necessity of the ASD diagnosis, although physicians often think ASD is not understood sufficiently within a “wide” view on a child. For example, an interviewee says:

I do not deny it [ASD], but for me it has to be part of a wider therapeutic process where things are not viewed narrowly. (physician 14).

While an ASD diagnosis is experienced as applicable to a very heterogeneous group of children, physicians often state that there is some central characteristic to ASD or that they can “feel” its presence. For example, the following physician says:

ASD, I think it is a feeling, a contact. Wow, that is, yes, I think that is very difficult to say. The way I feel a child mostly, yes, this is difficult to put into words. (physician 10).

The IQ is considered to have a big influence on the phenotype of ASD. Many physicians also repeatedly make a distinction between “real” or “nuclear autism” on the one hand, and ASD on the other; they frequently have different views on these two notions. The perceived differences concern the intellectual ability, the severity of the child’s problems, the visibility of his/her problems (i.e., to everyone, including professionals), and the evolution when he/she gets older. The list of *DSM* criteria is the only formal text on ASD that these physicians frequently refer to (6). In *DSM-5*, its current version, all types of autistic conditions [including Asperger’s syndrome and pervasive developmental disorder-not otherwise specified (PDD-NOS)] are merged into the category of ASD. Some physicians mourn this loss of distinction and find it more difficult to work with the broad term of ASD:

They have thrown it into one disorder [in creating DSM-5], but for me that is wrong. ‘Nuclear’ autism really is quite heavy. (physician 16).

And another physician:

In recent years, they have taught us to call everything ‘autism spectrum,’ while earlier, ‘autism’ was for children who really experienced problems in every one of the domains [of the DSM-criteria]. (physician 15).

#### Difficult Integrations

All interviewed physicians explicitly or implicitly say they use the *DSM* to guide the ASD diagnostic process. However, they experience the different sources of expertise on ASD as conflicting and difficult to integrate into some uniform view. The following physician says at the start of the interview:

Autism and ASD are perhaps difficult to explain, I will tell it as I feel and understand it, by training and study, and from my own experience, how I try to figure out what this is. (physician 13).

The difficult integration is experienced in three ways. First, some aspects of physicians’ views on ASD are transmitted during their training and when reading literature; other aspects spring from their personal clinical experience. These two sources of expertise sometimes clash: physicians say that what they have been taught during their residence is difficult to reconcile with what they have since learned from their own experience in the clinic. An interviewee says that during her training, ASD was transmitted to her as a fixed disability, while in her years of practice, she has gradually come to think of ASD as a dynamic condition that changes over the years:

During my training, ASD was presented as a handicap to me, but I made a switch afterwards; I now view ASD as changing over time. (physician 4).

Another interviewee talks explicitly about evidence-based medicine:

I do not have anything against ‘evidence-based’ and ‘best-practice guidelines’ but yes, this has become dogmatic, also directed by policy, and this frightens me in some way. I know I am not the only one: one has to put this ‘evidence’ and these ‘guidelines’ into perspective. I am glad there is scientific research, I am the first to ask for more research. But the clinical practice … we do not look at people to discover whether they meet the inclusion criteria; people who enter here, that is complex, you know. (physician 13).

Importantly, this is the only participant who talks about formal practice guidelines. As a second difficult integration for arriving at a uniform view on ASD, several physicians say that they have to reconcile different stances linked to their medical training compared to a possible psychotherapy training. Indeed, most of the participating child psychiatrists had psychotherapy training during or after their medical training. This child psychiatrist sets out how this difficulty affected her:

The context in which we were trained [medically] was very occupied with describing all these behavioral traits, while in the therapeutic approach, it was all about the ‘emerging feeling of self’ [cf. Daniel Stern]. I remember that this was really a breaking point for me, I was not able to make sense of this. The way teachers talked, it was so mechanistic for me, you really did not get at the inner person, this essential feeling of self, that you need to get in a relationship with people, to enter into contact. (physician 14).

A third and final difficult integration consists of physicians sometimes expressing how hard it is to meet certain external societal demands that need to be kept in balance with the experienced quality of care for their patients. They talk about the societal coercion they feel to give a formal diagnosis in order to obtain services for child and parents. One interviewee says:

You start to conform to … you lose your “polyphony.” You become bound to rules, thinking “What do I need to put in the report later on [after the consultation] in order to get a certain type of care or help?” (physician 14).

Indeed, for the physicians, the quality of care is always the most important aspect of their work.

### The Autism Spectrum Disorder Diagnosis Is a “Descriptive” Part of a Clinical Trajectory

Many physicians viewed an ASD diagnosis as a descriptive part of the clinical trajectory with the child and her family. The physicians often express a preference for what some interviewees call “process–diagnostics,” a therapeutic process in which a diagnostic assessment can be included, but only if useful—or necessary for acquiring certain entitlements:

We always do ‘process–diagnostics’. We are always able to explain that, especially with young children, we are very careful with this diagnosis [of ASD]. Often parents appreciate this, also because it leaves space and because you give the impression of ‘following things up.’ (physician 5).

However, some physicians indicate they do value a delineated formal diagnosis in the beginning of the clinical trajectory.

Sometimes the physicians find a diagnosis counterproductive in their clinical trajectory with a family. Complementary to or even instead of giving a formal classificatory diagnosis, they often express a preference for elaborating a “descriptive” diagnosis or a “profile” of the child. They prefer to describe the entire person of the child with his/her strengths and weaknesses:

For me, everybody has his strengths and weaknesses, and ASD is just a weakness, nothing more than that. What is the context? What are the child’s other competences to compensate for this weakness? And I believe these elements are much more important than the fact that this [the ASD diagnosis] is written on paper. (physician 3).

Accordingly, the interviewed physicians frequently question the possibility to use ASD as a categorical diagnosis. They often say that ASD is difficult to define and diagnose, especially in young children, and that ASD is difficult to differentiate from other diagnoses. Several physicians experience the diagnostic criteria of ASD as subject to personal interpretations and rather arbitrary cutoffs.

It always is a weighed diagnosis, you know, the [physician’s] personal estimate. In my case this personal cutoff is as high as you could … I am not going to problematize things too quickly (physician 12)

Also, physicians often report that the facts about ASD are unclear and do not provide them with clear messages (for example, about causes and prognosis) that they can convey to parents coming to them for help with their children:

I always tell parents: “Look, we do not know much about it.” (physician 4).

Nevertheless, several physicians express the opinion that it is possible to correctly diagnose ASD in children.

Although I believe it is not wise to do a diagnostic assessment during a crisis, often the diagnosis turns out to be correct. (physician 13).

Moreover, we only found one participant who talked about diagnostic testing tools, suggesting that tests do not carry much weight in the physicians’ diagnostic decision. More often, these physicians say that they rely on multidisciplinary collaboration and information from the context to decide on a diagnosis:

The story of parents is very valuable, it is certainly taken into account. (physician 8).

Besides, apart from the parental role in the diagnostic assessment, many physicians are convinced about the parents’ influence on the ASD’s phenotype and evolution. This physician tells how her concerns about a child with ASD are dependent on the parents:

If a child comes from a good home, then I know I can rest assured. (physician 4).

During the diagnostic assessment, some physicians also emphasize what the child is saying through his/her behavior:

[Talking to parents]: “What does your child want to say with her behavior? Behavior is a way to communicate. How can I read and understand my child? And how can I relate to this?” (physician 13).

### Autism Spectrum Disorder Treatment Is a Mix of “Standard” Approaches and a Personalized Search

The third theme concerns physicians’ views on the treatment of ASD. In their work, the physicians are found to value *care* most: diagnostic assessments always have to be in the service of the care they wish to provide to the child and his/her family. It is remarkable that the physicians do not mention any typical ASD-specific child-directed interventions in the interviews like Early Intensive Behavioral Intervention (EIBI). In the families with a diagnosed child, they advise parental guidance (from themselves and other professionals), and, at school, help for teachers to adapt the classroom and teaching situation to the child. All physicians say that they adhere to what interviewees sometimes call the “standard” measures of what children with an ASD diagnosis need from their environment, mentioning structure, predictability, and (only once) visualization. These strategies are experienced as useful, despite the diagnosis being difficult to define and deliver:

These are the general standard things, I am acting in a different way. (…) I make sure that things are predictable, so I install a framework because it provides calmness for the child, so that we can then look at “What is your child trying to say?” (physician 4).

The participants have the experience that the “standard” measures are also useful for other children, with no ASD diagnosis:

I think this is soothing for a great many children. (physician 2).

Moreover, they are convinced that these “standard” measures always need to be supplemented with a personalized search for what works for an individual child:

To make a fundamental difference for children with ASD in the same way as in ADHD, I have seen this less frequently. If I give it the name ASD or not, the child, e.g., has problems with her classmates, so we will still have to find out what we can do about this. (physician 12).

In this personalized search, physicians often stress the importance of the parents’ experiences of their child-rearing practice, and sometimes of the communication through the child’s behavior.

Maybe that is also very important for parents, that they know how to handle their child. I think it is very good to empower parents. (physician 5).

And another physician:

[Talking to parents] “We will try to understand what your child wants to say with this behavior.” (physician 4).

Thus, just as the diagnosis is often experienced as one part of the (“profile” of a) child, what is considered to be the “standard” approach linked to the diagnosis is also viewed as providing one part of the necessary treatment.

## Discussion

This study demonstrates that what is experienced as a “textbook knowledge”^6^ view on ASD is only one part of physicians’ views on ASD. Indeed, previous studies have found many “gaps” between official values and codes of conduct, and those adopted within day-to-day practice (52). Not only is the evidence concerning ASD heterogeneous and difficult to synthesize, we found that ‘evidence-based medicine’ is not the only thing to guide the interviewed physicians when dealing with ASD in their practice with young children without ID and other disabilities. Indeed, Ghaemi argues that “because most psychiatric illnesses are complex phenomena, no single method or approach is sufficient to explain them or the experiences of persons who suffer from them” (53). Accordingly, warnings are proffered against accepting research conclusions that oversimplify practitioner conceptualizations of illness (50).

Moreover, concerning the registered differences between different clinicians, broad differences have been argued to exist between the ways in which psychiatrists understand and treat mental illness^3^ (Schatzman and Strauss, 1966), even while their work and discourse are grounded within (inter)nationally shared training, governance, and regulation (51). In our study, this “grounding” is reflected by the fact that the neurobiological reality of ASD is not doubted by any of our interviewees. Nevertheless, these physicians work to integrate what they experience as “textbook knowledge” concerning ASD with both their general clinical styles (i.e., beyond ASD) and their clinical experience with ASD. The heterogeneous facts on ASD seem to leave enough space for interpretation in order to reconcile knowledge, experience, and clinical attitude in a very personal way. Importantly, this personal synthesis is reported by the participants to be workable in their clinical practice. This finding resonates with the concept of ‘adaptive expertise’ which in research on education has been devised to point to how teachers solve tasks by adaptive and (meta)cognitive skills like an epistemic stance that views the world as complex (54).

The interviewees experience the *DSM* as an important guide in the diagnosis of ASD, and indeed the *DSM* has been described as a prominent and powerful tool for a range of actors (in the mental health domain), and its importance “cannot be overstated” (51). Though clearly integral to contemporary mental health, psychiatrists have been found to work with the *DSM* in a variety of ways, reconfiguring its categories to fit with existing work practices, and personal and professional attitudes and approaches (51). We found evidence that this statement about the *DSM* can be broadened to apply to “textbook knowledge” in general—at least for our population of physicians working with preschool children without a disability diagnosis and with (a presumption of) a diagnosis of ASD. Moreover, it has been argued that perhaps standardized diagnostic manuals (as *DSM*) delegate medical science to clinicians, and that many of these manuals fail to become correctly ‘configured’ in the face of the patients that clinicians see (55). Indeed, there continues to be a tension in the application of classifications at the local clinical level (21). Clinical staff try to merge different approaches, but as there are no coherent syntheses, they appear to be left to their own devices in working out how such a merger might take place (21).

Another integration in the interviewees’ view on ASD is made between a perspective on ASD as a neurobiological reality and a perspective on ASD as influenced by psychological and social factors. As such, we provide empirical evidence concerning ASD in young children without disability for the claim by Barrett: “Even when neurological models are invoked to explain psychiatric disorder, they may be employed alongside an assortment of other perspectives, as psychiatrists move between differing biological, psychological and social understandings of psychopathology as part of a ‘biopsychosocial’ model of theory and practice” (51, 56).

The physicians in this study often find ASD difficult to define, diagnose, and explain clearly to parents. Only one physician mentions diagnostic tools (although all the centers these physicians work in are known to be using tools). This one physician said that tools were not really helpful to diagnose ASD with certainty, because the test scores were regularly overruled by a clinical intuition in the team. Previous research has highlighted the narrative process of diagnosing ASD in teams (57, 58). Our interviewees value contextual information and a multidisciplinary diagnostic assessment. It is indeed known that the most effective way to organize and deliver ASD services is through multidisciplinary teams (8, 50, 59). Also, the interviewed physicians regularly do not think an ASD diagnosis is useful and necessary at the accepted moment, i.e., at the beginning of a clinical trajectory (60). In addition to a classificatory diagnosis, they regularly express a preference for a “descriptive” diagnostic profile and for what some of our interviewees call “process–diagnostics,” a therapeutic trajectory in which a diagnostic assessment can be included, but only if they consider it useful—or necessary to acquire certain entitlements (for an elaboration of the implications of an ASD diagnosis experienced by these physicians, like acquiring entitlements, see the report of the other half of this study’s results) (35). They believe that by working this way they can provide better care to their patients. In line with this finding, clinicians have previously been found to think that formal diagnostic requirements can conflict with patient-care objectives (21). In mental health in general, a “clinical case formulation” has been recommended to identify a broad range of idiosyncratic aspects of a clinical case and to select interventions (61). In a shared decision-making model, however, both such a diagnostic “formulation” and the decision, at a certain point in the clinical trajectory, to apply the categorical diagnosis of ASD would be made in dialogue with the patient–parents in this case.

When it comes to the treatment offered to families with a child with an ASD diagnosis, the participants do not mention any ASD-specific child-directed interventions like EIBI, but it is worth noting that these treatment modules are not readily available in Flanders for children without ID ^5^. The “standard” approach—as perceived by our physicians, i.e., mainly structure and predictability—is valued but is seen as nonspecific and as having to be supplemented by personalized approaches. It has indeed been described that, concerning mental disorders in general, a disparity exists between explicit “textbook” principles and the implicit ideas and assumptions employed by professionals during clinical practice (50). Although personalized medicine is on the rise in psychiatry, it is often viewed in a biological way (14, 62, 63), though not always (61). The physicians we interviewed express how with ASD, one has to search for “what works for whom,” not only in a biological sense, but also in a psychological sense. A similar mandate for individualizing psychotherapy was embodied in Gordon Paul’s (1967) iconic question: “*What* treatment, by *whom*, is most effective for *this* individual with *that* specific problem, and under which set of circumstances?” (64). And so, these physicians’ views on ASD treatment often seem to be as much in line with psychotherapeutic as with biomedical approaches.

These findings confirm and add to what has already been ascertained about physicians’ conceptualizations of ASD in research ^1^. Our results enable a drawing of an encompassing picture of how physicians view and experience ASD, the ASD diagnosis, and its clinical use for helping and caring for a child and her parents. The clinical translation and application of research results on ASD appears to not be straightforward, and therefore, merits more attention in future research. In this translation indeed, a clinical approach inspired by findings from most current research on ASD (13) may be only partially helpful to physicians in their clinical practice. In order to be “workable” in their clinical practice, these physicians appear to need diagnostic categories that they can reconcile with both their personal clinical style and the needs of parents and child.

Our study has several limitations. This interview study was conducted with physicians who volunteered to participate, and they were all eager to talk about their views on ASD. Thus, there may have been self-selection bias. All candidates were experienced clinicians (so, not researchers). Their training backgrounds were variable, but as a group they represented the different physicians working with young children with a (presumed) ASD diagnosis in Belgium. Since this is a qualitative study, the aim is not to quantify the opinions of our participants. This study was conducted in Flanders, but with an appropriate contextual translation, the results can shed light on clinical ASD work in other regions and cultures. In addition to interviewing physicians working with young children, we are also interviewing parents of young children with an ASD diagnosis and adults with a diagnosis. Of course, a valuable next step will be to query older children and adolescents diagnosed with ASD.

## Conclusions

In summary, ambiguities are found to be inherent when dealing clinically with ASD, yet they might not excessively interfere with a physician’s work. The interviewed physicians are confronted with several difficult integrations, between the “textbook knowledge” on ASD and their clinical experience, between their medical and their psychotherapeutic clinical stance, and between the societal demands for a diagnosis and their aim to provide good care to their patients. These physicians deal with these ambiguities and difficult integrations by embedding their eclectic view within their personal clinical styles, and by selecting from the “textbook knowledge” what works for them, and for the parents and child.

## Ethics Statement

This study was carried out in accordance with the recommendations of the internal guidelines of the Ethics Committee of the University Hospitals Leuven with written informed consent from all subjects. All subjects gave written informed consent in accordance with the Declaration of Helsinki. The protocol was approved by The Ethics Committee of the University Hospitals Leuven on 3 February 2017 (Belgian Registration Number B322201731147). The study was performed in accordance with the ethical standards laid down in the 1964 Declaration of Helsinki and its later amendments.

## Author Contributions

DJ, JS, KD, and KH were all involved in the design of the study. DJ performed the data collection and analysis and wrote the first draft. DJ, JS, KD, and KH all participated in further analysis and fine-tuning of the themes emerging from the analysis. DJ wrote the further drafts of the paper, which were commented on by JS, KD, and KH. All authors read and approved the final version of the paper.

## Funding

This research was funded by the Leuven University Fund, grant “Opening the Future.”

## Conflict of Interest Statement

The authors declare that the research was conducted in the absence of any commercial or financial relationships that could be construed as a potential conflict of interest.

## Footnotes

1 Jacobs D, Hens K, Steyaert J, Dierickx K. The conceptualization of autism by parents and clinicians—a review of empirical qualitative studies. under review.

2 It was impossible to neatly distinguish physicians’ experiences of ASD and of an ASD diagnosis, since the *DSM* they were using explicitly defines what ASD is by enumerating the criteria that have to be fulfilled in order for a person to merit an ASD diagnosis. Thus, the two concepts are inseparable.

3 The *exact objective degree* of experience was judged to be irrelevant to our research question on a physician’s experience and view of ASD. *Subjectively*, all these physicians felt qualified to say relevant things on the topic.

4 The authors of this study do not specify how they defined a “specialized outpatient clinic,” and do not report which specific types of specialized physicians were working in specialized outpatient clinics and rehabilitation centers. In our experience, in these two types of centers, child psychiatrists, child neurologists, disability physicians, and pediatricians are working with i.a. the clinical population of this study, i.e., children younger than 6 years of age without a disability diagnosis.

5 Steyaert J. Personal communication.

6 We use the concept “textbook knowledge” as meaning “(inter)nationally shared training, governance and regulation” (50, 51) in order to capture our interviewees’ implicit understanding of what they consider as ‘official’ knowledge on ASD.
